# Sublingual buprenorphine for acute renal colic pain management: a double-blind, randomized controlled trial

**DOI:** 10.1186/1865-1380-7-1

**Published:** 2014-01-03

**Authors:** Pooya Payandemehr, Mohammad Jalili, Babak Mostafazadeh Davani, Ahmad Reza Dehpour

**Affiliations:** 1 Emergency Medicine Department, Imam Hospital, Tehran University of Medical Sciences, Keshavarz Blvd, Tehran, Iran; 2 Tehran University of Medical Sciences, Keshavarz Blvd, Tehran, Iran; 3 Department of Pharmacology, School of Medicine, Tehran University of Medical Sciences, Poursina St, Keshavarz Blvd, Tehran, Iran

**Keywords:** Buprenorphine, Emergency department, Pain management, Renal colic

## Abstract

**Background:**

The aim of this study was to compare the efficacy and safety of sublingual buprenorphine with intravenous morphine sulfate for acute renal colic in the emergency department.

**Methods:**

In this double-dummy, randomized controlled trial, we enrolled patients aged 18 to 55 years who had a clinical diagnosis of acute renal colic. Patients received either 2 mg sublingual buprenorphine with an IV placebo, or 0.1 mg/kg IV morphine sulfate with a sublingual placebo. Subjects graded their pain with a standard 11-point numeric rating scale (NRS) before medication administration and 20 and 40 minutes after that. The need for rescue analgesia and occurrence of side effects were also recorded in the two groups.

**Results:**

Of 69 patients analyzed, 37 had received buprenorphine, and 32 had taken morphine. Baseline characteristics were similar in both groups. NRS pain scores were reduced across time by administration of both buprenorphine (from 9.8 to 5.22 and then 2.30) and morphine (from 9.78 to 4.25 and then 1.8), significantly (*P* <0.0001). The two regimens did not differ significantly for pain reduction (*P*?=?0.260). Dizziness was more frequently reported by the buprenorphine group (62.1% versus 37.5%, *P* <0.05) but other adverse effects observed within 40 minutes were similar in the two groups.

**Conclusions:**

Sublingual buprenorphine (2 mg) is as effective as morphine sulfate (0.1 mg/kg) in acute renal colic pain management.

## Background

Renal colic is a severely painful condition frequently encountered in emergency departments (EDs) [1]. As relieving the patient’s pain and suffering is one of the first responsibilities of emergency physicians, finding safe and effective methods to accomplish this task is of paramount importance [2]. The routine practice for pain reduction in renal colic is intravenous (IV) administration of analgesics, either non-steroidal anti-inflammatory drugs or opioids [3]. IV administration of these drugs, although effective, takes a lot of staff time, which is not ideal in constantly overcrowded EDs. On the other hand, 'time to analgesia’ is an indicator of ED service quality, which will be much longer when using a parenteral route for analgesia [4]. The ideal analgesic drug is one with enough efficacy and the fewest side effects as well as easy route of administration.

Buprenorphine is an analgesic with mixed agonist-antagonist properties which has been shown to have a role in acute pain management in postoperative pain and orthopedic injuries [5,6]. However, there is only one study evaluating its effectiveness in renal colic [7] and, to the best of our knowledge, there is no study using the sublingual form of buprenorphine as an analgesic in renal colic. Its sublingual form can be administered rapidly and with no need for IV lines, and hence may be an ideal drug for acute pain management in renal colic.

We compared the effectiveness of sublingual buprenorphine with that of IV morphine sulfate for pain management in ED patients with renal colic.

## Methods

### Study design

This is a double-dummy, placebo controlled randomized clinical trial in patients with acute renal colic in the ED. Using block randomization (with a computer generated sequence) and a sealed envelope mechanism for allocation concealment, patients were divided into two groups, one receiving sublingual buprenorphine with IV placebo and the other receiving IV morphine with sublingual placebo. The study was performed in the emergency department of Imam Hospital, a tertiary referral center with approximately 50,000 visits per year. The ethics committee approved the study protocol and the study was registered in http://www.clinicaltrials.gov (NCT01546701).

### Study setting and population

Eligible patients for our study were those with acute colicky pain aged between 18 and 55 years old and who had a clinical diagnosis of acute renal colic based on medical history and a positive urinalysis for hematuria. The numerical rating scale (NRS), validated by Bijur et al., was used to measure severity of pain [8]. All patients with a pain score of more than 3 who signed the informed consent, were enrolled in the study. We excluded patients with previous history of seizure; cardiovascular, hepatic, renal or metabolic diseases; febrile patients (T >38°C); hemodynamically unstable patients (systolic blood pressure <90 mmHg); and pregnant patients. We also excluded patients with abdominal tenderness as a sign of peritoneal inflammation and those with any clinical suspicion for diseases other than urolithiasis, including abdominal aortic aneurysm or dissection. Patients with a history of drug addiction or known allergy to opioids and those who had received analgesics 6 hours before arriving at the ED were also excluded to prevent any possible drug reactions.

### Study protocol

Eligible patients were randomly allocated into two groups to receive either sublingual buprenorphine as 2 mg tablets plus IV injection of 0.1 mL/kg sterile water or a sublingual placebo plus 0.1 mg/kg IV morphine sulfate (1 mg/mL). All the researchers and participants remained blind to the treatment and results throughout the study.

### Outcome measures

The primary outcome was the efficacy of the drug with regards to reducing the NRS 20 and 40 minutes after administration and the need for rescue analgesia, which was an IV injection of fentanyl (0.75 μg/kg). The secondary outcome was the occurrence of adverse effects.

### Measurements

Patients were asked to rate their pain in a verbally administered NRS with 0 representing no pain and 10 the worst imaginable pain. The severity of pain was reevaluated at 20 and 40 minutes after analgesic administration. The patients were monitored for any change in their vital signs including blood pressure, pulse rate, respiratory rate and oxygen saturation during the study. Hypotension was defined as a drop of more than 20 mmHg in systolic blood pressure, and respiratory depression as a respiratory rate below 12 (if it was initially more than 12 per minute). The patients were also asked about their feelings of nausea, vomiting, dizziness, pruritus, or drowsiness.

### Data analysis

Baseline demographic and clinical variables were compared using independent Student’s *t*-tests or exact tests. Repeated measure ANOVA was used to assess the effect of intervention on pain scores across time and between the study groups at each time point. Side effects were compared using χ^2^ tests and *P* <0.05 was considered statistically significant. The sample size was calculated as 25 for each group according to the study conducted by Finlay et al. [7] with α and β error being 0.05 and 0.20, respectively. We enrolled 80 patients to cover possible withdrawals from the study. All analyses were performed using the Statistical Package for Social Science version 15 (SPSS Inc., Chicago, IL, USA).

## Results

### Patient characteristics

**Figure 1:**
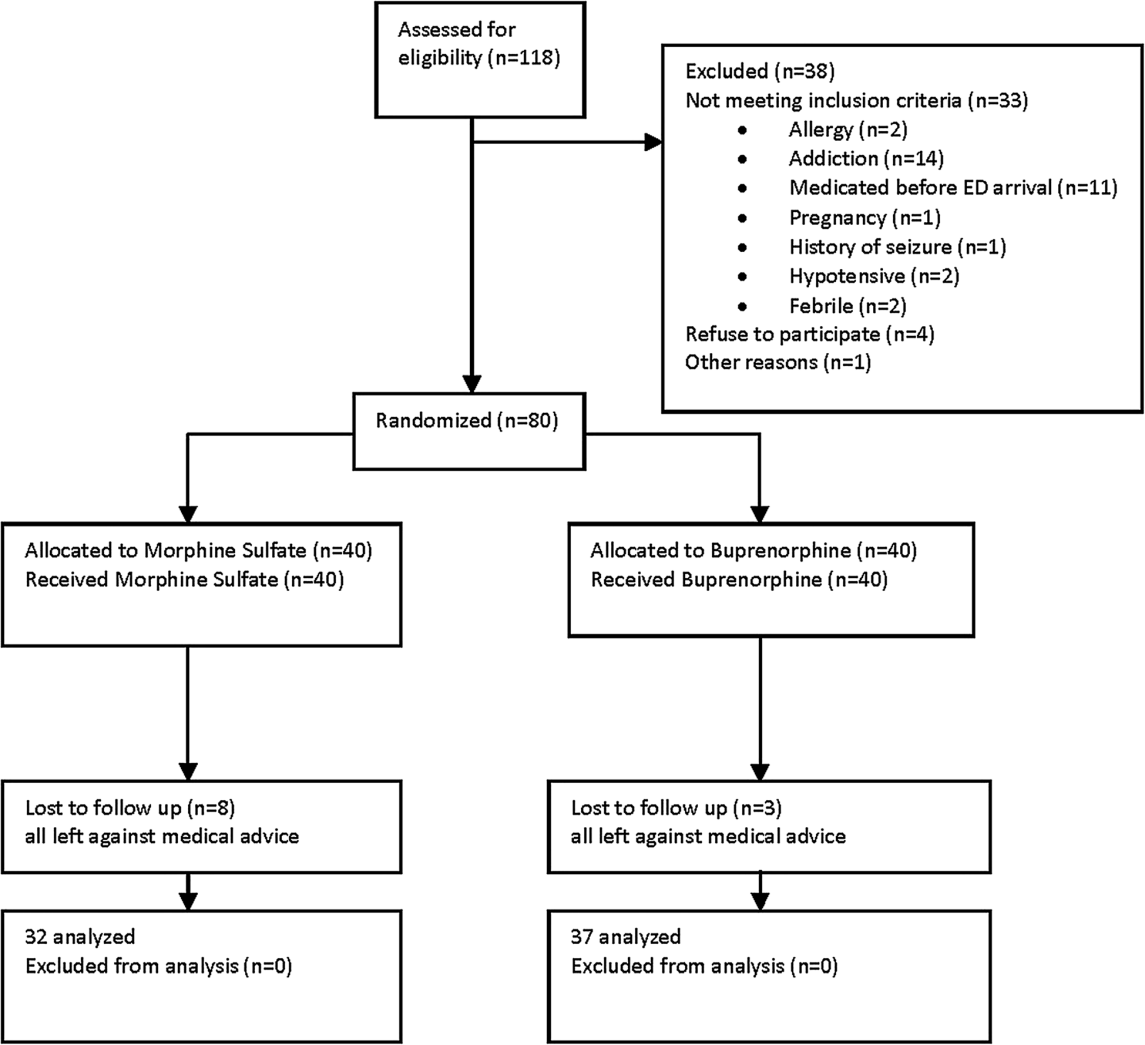
Flow of participants through trial.

Between March 2011 and April 2012, 118 patients with acute renal colic were assessed for eligibility, 38 were excluded from randomization because they did not meet the inclusion criteria and 80 were enrolled and randomized into the two treatment groups. During the study period, 11 patients were lost to follow-up. Of 69 patients whose data were finally analyzed, 37 and 32 received buprenorphine and morphine, respectively (Figure 1). Baseline characteristics were similar in the two groups (Table 1).

**Table 1:** Baseline demographic and clinical characteristics of study participants

**Group**	**Buprenorphine n?=?37**	**Morphine n?=?32**
Female (%)	9.84 (±0.50)	9.78 (±0.55)
Mean age (year)^*^	35 (±10)	31 (±10)
Respiratory rate (per minute)^*^	13.38 (±1.55)	13.13 (±1.28)
Pulse rate (per minute)^*^	90.95 (±9.40)	94.63 (±11.48)

*Values in mean (±SD).

### Primary outcome

**Figure 2:**
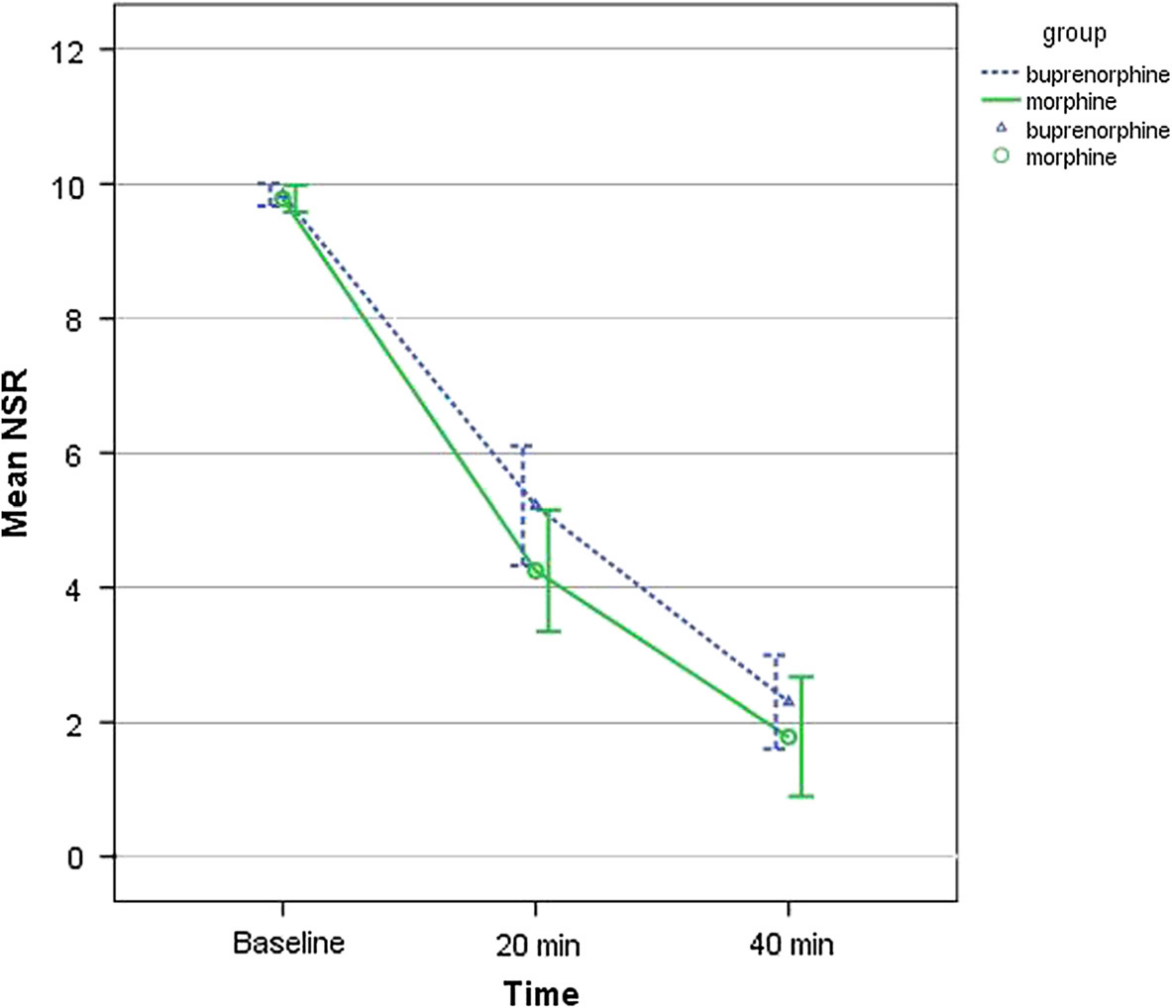
NSR pain score over the study period.

There was no significant difference between NRS scores of the two study groups at the beginning of the study. The NRS pain scores showed a significant reduction across time (*P* <0.001) when analyzed using the repeated measure ANOVA in both groups (Table 2). The two groups did not differ significantly for pain reduction (*P*?=?0.260) (Figure 2). The use of rescue analgesia was not significantly different in the two groups (Table 2).

**Table 2:** Pain scores in study groups

**Group**	NRS0*	NRS20*	NRS40*	Rescue analgesia**
**Buprenorphine**	9.84 (±0.50)	5.22 (±2.66)	2.30 (±2.09)	5 (13.5%)
**Morphine**	9.78 (±0.55)	4.25 (±2.51)	1.78 (±2.45)	2 (6.25%)

*Mean numerical rating score (±SD) at baseline and after 20 and 40 minutes.

**Number of patients who needed rescue analgesia after 40 minutes.

### Secondary outcome

There was also no significant difference between the study groups regarding patients’ vital signs after administration of the medications except for the respiratory rate 20 minutes after treatment, which was significantly lower in the morphine group (mean 11.41 bpm vs. 12.27 bpm) (*P*?=?0.017). No significant difference between the two groups was noted in terms of respiratory depression and hypotension. There was no significant difference between the two groups regarding the occurrence of nausea, vomiting or pruritus, except that the patients in buprenorphine group reported dizziness more frequently (*P* <0.05) (Table 3). There were no cases of seizure or loss of consciousness in either group.

**Table 3:** Occurrence of side effects in the study groups

**Group**	**Nausea**	**Vomiting**	**Drowsiness**	**Pruritus**	**Dizziness**	**Respiratory depression**	**Hypotension**
**Buprenorphine**	20 (54%)	4 (10.8%)	6 (16.2%)	0 (0%)	23 (62.1%)*	10 (27%)	17 (45.9%)
**Morphine**	20 (62.5%)	3 (9.3%)	6 (18.7%)	1 (3.1%)	12 (37.5%)	11 (34.3%)	15 (46.8%)
***P *** **value**^†^	0.47	1.000	0.782	0.464	0.041	0.508	0.938

*Statistically significant (*P* <0.05).

^†^χ^2^ test, Fisher’s exact test.

## Discussion

The results of this study demonstrated that administration of 2 mg sublingual buprenorphine effectively reduced pain in patients with acute renal colic. Although the number of patients was not enough to perform equivalency tests, the results showed that the analgesic effect of buprenorphine was comparable to 0.1 mg/kg IV morphine.

To the best of our knowledge, there are no other studies on sublingual buprenorphine in the treatment of renal colic in the ED. However, our results are compatible with the results of a previous study in this center on the effectiveness of sublingual buprenorphine in acute bone fractures [6]. In that study, patients with acute extremity fractures received either sublingual buprenorphine (0.4 mg) or IV morphine (5 mg). The pain scores were compared after 30 and 60 minutes and there was no significant difference between the two groups [6]. Our results are also comparable with the results of Risbo et al. [9] and Abid et al. [10], who studied buprenorphine in post-operative pain management. In the first study, buprenorphine was compared with intramuscular morphine as an analgesic for pain management after elective knee joint surgery, where they demonstrated similar efficacy [9]. In the second study, Abid et al. found buprenorphine as effective as morphine for post-operative pain management in patients undergoing a Caesarean section [10]. Furthermore, according to the findings of this study, patients in the buprenorphine group reported dizziness more frequently but other adverse effects observed within 40 minutes were similar in the two groups. Walsh et al. [11] showed that increasing buprenorphine dose will increase its analgesic effect but not its side effects. Comparing to the results of a study in which lower dose of buprenorphine was used (0.4 mg) and no adverse effect was reported [6], it seems that higher doses will increase analgesic efficacy without affecting the side effects.

### Limitations

Our study faced several limitations. First, many patients had to be excluded due to previous use of opioids or addiction. Secondly, some of the patients might have been drug seekers and IV drug users imitating renal colic to get morphine. It is possible that they were not recognized and were included in study. Thirdly, NRS may be an inappropriate measurement upon arrival of the patients as they tend to choose the highest NRS (i.e., 10), assuming that this will accelerate their treatment process. It may justify the number of patients whose NRS was 10 at the beginning; however, this situation is expected to be similar in both groups and does not seem to influence the change in NRS during the observation time. Another limitation of our study was the use of 2 mg buprenorphine tablets which may be the cause of slightly higher rate of side effects in this group; using smaller doses and then titrating it to effect may reduce the occurrence of side effects. Finally, this study did not find a significant difference between the two groups probably because of the small sample size.

## Conclusions

In conclusion, sublingual buprenorphine can be used as an effective analgesic with minor side effects for acute pain relief in patients with renal colic, and its effectiveness is comparable to that of IV morphine.

## Abbreviations

ED: Emergency department; IV: Intravenous; NRS: Numeric rating scale.

## Competing interests

All authors declare that they have no competing interests.

## Authors’ contributions

PP and MJ developed the study questions, designed the methods for data acquisition, and supervised the collection of data. BMD performed statistical analysis. PP, MJ, and BMD jointly participated in drafting the manuscript. AD helped in the interpretation of data and also revised the manuscript critically. All authors read and approved the final version of the manuscript.
